# Prevalence of degenerative alterations of osseous temporomandibular joint structures in cone beam computed tomograms in a consecutive sample of young adults. A retrospective study

**DOI:** 10.1007/s00784-025-06541-9

**Published:** 2025-10-21

**Authors:** Dagmar Schnabl, David Pamminger, Sebastian Rohde, Linus Hupp, Ansgar Rudisch, Rüdiger Emshoff

**Affiliations:** 1 University Hospital of Dental Prosthetics, Medical University of Innsbruck, Innsbruck, 6020 Austria; 2 University Hospital of Oral and Maxillofacial Surgery, Medical University of Innsbruck, Innsbruck, 6020 Austria; 3 Department of Radiology, Medical University of Innsbruck, Innsbruck, 6020 Austria

**Keywords:** Condylar erosion, Imaging, Orthodontics, Temporomandibular joint osteoarthrosis

## Abstract

**Objective:**

To assess the prevalence of bony temporomandibular joint (TMJ) alterations in cone beam computed tomography (CBCT) scans in young adults.

**Materials and methods:**

CBCT images (taken in the years 2017 to 2022) of consecutive patients aged 18 to 30 years, who had skull CBCT prior to the removal of wisdom teeth, were evaluated by one experienced examiner with regard to degenerative osseous TMJ findings. Records of patients diagnosed with TMJ disorders were excluded. Respective orthopantomograms were screened for the presence of fixed orthodontic retainer wires hinting at previous orthodontic treatment.

**Results:**

CBCT scans of 213 patients (mean age 23.9 ± 3.5; 135 women, 78 men) were evaluated. Bony alterations in both TMJs were found in 39.9% and bony alterations on at least one side in 64.3%. Of 213 patients, 41.3% had condylar erosions, 28.2% condylar flattening 24.4% condylar osteophyte formation, 16% condylar subchondral sclerosis, 4.7% condylar resorption, 3.8% condylar subchondral cysts, and 8% fossa sclerosis on at least one side. The presence of orthodontic retainer wires, present in 55 patients, increased odds for condylar erosions in at least one TMJ by 4.046 (*p* < 0.001).

**Conclusions:**

Degenerative osseous TMJ changes are a frequent finding in young adults. Orthodontic treatments seem to impact the prevalence of osseous TMJ alterations.

**Clinical relevance:**

Future studies should clarify the clinical relevance of osseous TMJ alterations and investigate their pathogenesis and pro-/regression with the passing of time in the context of diverse malocclusions and orthodontic measures.

## Introduction

The presence of imaging signs in osseous temporomandibular joint (TMJ) structures has been used as a criterion in the classification of temporomandibular dysfunctions (TMDs) that may differentiate simple arthralgia from osteoarthritis and, in the absence of pain, confirm the diagnosis TMJ osteoarthrosis [1].

**Fig. 1:**
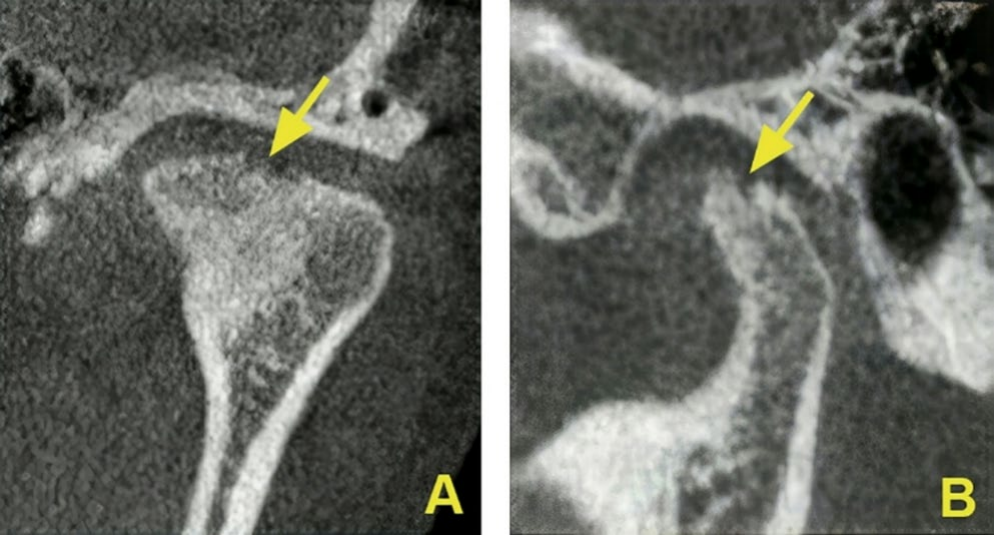
CBCT sections displaying condylar erosion. (**A**) Coronal plane. (**B**) Sagittal plane

Osseous changes of the condyle comprise (1) flattening, defined as a flat bony contour deviating from the convex form; (2) erosion, defined as an area of decreased density of the cortical bone and the adjacent subcortical bone (example, Figs. 1 A and B); (3) osteophyte formation, defined as marginal bony outgrowths on the condyle; (4) sclerosis, defined as an area of increased density of cortical bone extending into the bone marrow; and (5) resorption, defined as partial loss of condylar head [2]. Osseous changes of the mandibular fossae subsume (1) erosion, (2) sclerosis, and (3) resorption [2].

According to a previous classification of osseous TMJ signs, condylar erosions present the least severe alteration (type 1), followed (in order of increasing severity) by condylar flattening (type 2), condylar deformity (type 3), condylar sclerosis (type 4), ankylosis (type 5), erosion of the fossa/eminence (type 7), and sclerosis of the fossa or eminence (type 8) [3]. By contrast, the Diagnostic Criteria for TMDs (DC/TMD) consider flattening and/or cortical sclerosis indeterminate findings that may represent normal variation, aging, remodeling, or a precursor to degenerative joint disease (DJD). The presence of at least one of the following signs is, however, diacritic in the diagnosis of DJD: Subchondral cyst(s), erosion(s), generalized sclerosis, or osteophyte(s) [4].

Bony alterations constitute an advanced stage in the pathophysiological course of the gradual progressive destruction of articular tissues [5]. Previous CBCT studies revealed an association between degenerative osseous TMJ changes and painful TMJ osteoarthritis (including rheumatic systemic arthritis) [6–10]. Some investigations have compared the prevalence of degenerative osseous TMJ findings between individuals with TMD and individuals not presenting with TMD.

Cho at al. (2012) found a rate of 26.8% osteoarthritic changes in 362 joints of adolescents with TMD (mean age 15.6 years). In comparison, osseous changes (flattening, sclerosis, osteophyte, or erosion) were assessed in 9.9% of 202 investigated TMJs of a control group of asymptomatic adolescents and young adults (mean age 15.2 years), who underwent CBCT for the examination of tooth impaction or orthodontic evaluation [6].

Wang et al. (2013) found CBCT-radiographic TMJ alterations (mainly flattening and shortening of the condyle and sclerosis) in 12.1% of subjects of a control group (*n* = 339) of pre-orthodontic adolescents and young adults with malocclusion (without TMD) aged 10–19 years. Of the subjects (same age group) with TMD (*n* = 368), 40.7% were diagnosed with radiographic signs of osteoarthritis [7].

According to a recent scoping review, several studies have investigated the morphology and degenerative changes in the TMJ using CBCT prior to orthodontic therapy [11]. Skeletal and dental class II malocclusions may be risk factors for developing radiographically detectable degenerative changes and skeletal and dental class III malocclusions seem to contain a higher risk of TMD occurrence [11].

To the best of the authors’ knowledge, there are no CBCT studies that have investigated the effect of orthodontic therapy on TMJ morphology in young adults. This retrospective study was carried out to assess the prevalence of degenerative osseous TMJ findings in a consecutive sample of young adults, who had skull CBCT prior to the removal of wisdom teeth, and to compare the prevalence of degenerative osseous TMJ alterations between individuals without and individuals with orthodontic retainer wires hinting to previous (fixed) orthodontic treatment.

## Materials and methods

### Ethical Approval

The present study was conducted in accordance with the 1964 Declaration of Helsinki and its later amendments. Ethical approval was obtained prospectively by the Ethics Committee of the Medical University of Innsbruck, study number 1042/2022. The study was registered at the registry for clinical studies of the University Hospital of Innsbruck (Clinical Trial Center ctc.tirol-kliniken.at), registration number 20230327-3164.

### Subjects

Skull CBCT scans of consecutive patients aged 18 to 30 years, who had skull CBCT imaging at the University Hospital of Oral and Maxillofacial Surgery of Innsbruck between January 1 st 2017 and December 31 st 2022 for the purpose of treatment planning of the surgical removal of wisdom teeth, were included, if TMJs were in the field of view. In the course of the pre-operation assessment and discussion, patients were routinely inquired about orofacial pain, TMD, previous orofacial trauma, or systemic diseases. Exclusion criteria were (1) craniofacial syndromes; (2) craniofacial trauma related to the TMJ; (3) systemic medical conditions involving joints; and (4) history of TMD. The corresponding orthopantomograms (taken six months at the longest before the respective CBCT scan) were screened for the presence of fixed (post-) orthodontic mandibular and/or maxillary retainer wires.

### CBCT data acquisition

CBCT was performed in an upright position with teeth in habitual occlusion with the machine KaVo 3D eXam (KaVo Dental GmbH, Biberach, Germany) by use of the following scanning settings: Field of view 16 cm x 13 cm; voxel size 0.3 mm; scanning time 24 s; tube voltage 90 kV; tube current 8.0 mA; slice thickness 0.215 mm. Three-dimensional reconstruction was accomplished with dental software OnDemand3D (KaVo Dental GmbH).

### Radiodiagnosis

The CBCT images were interpreted by a single experienced medical clinician without knowledge of clinical findings/anamnesis or the presence/absence of orthodontic retainer wires (retrieved from orthopantomograms). Reliability scores were determined by administering the imaging criteria on a set group of images, thereby allowing for intra-rater comparison. Intra-examiner reliability was high (kappa = 0.9%; kappa statistics).

CBCT scans were evaluated with respect to the presence or absence of the following osseous changes that were visible in both sagittal and coronal sections: Condylar erosion (example, Fig. 1 A and B), condylar osteophyte, condylar subchondral sclerosis, condylar flattening, condylar resorption, condylar subchondral cyst formation, fossa erosion, fossa sclerosis, and fossa resorption [2].

### Data analysis

The power analysis for logistic regression (two-tailed) revealed a minimal sample size of 96 subjects based on the following assumptions: the prevalence of condylar TMJ osteoarthritis among orthodontically treated patients would be 18.8%, and 6.5% among asymptomatic controls [12], with an odds ratio of 3.12 (R^2^ other X = 0.2), an alpha error of 0.05, and a statistical power of 85%. For sample size estimation, the G*Power software (version 3.1) was applied[13].

Data were analyzed by descriptive statistics. Pearson’s chi-square test was used to assess differences between subgroups. Binary multivariate logistic regression analysis of influencing factors was applied to assess the association between the presence of erosions and orthodontic retainer wires. The significance level was set at *p* ≤ 0.05.

## Results

### Subjects

In total, 213 subjects (135 women and 78 men) at a mean age of 23.9 ± 3.5 years were included in the study.

### Prevalence of osseous alterations

Altogether 137 subjects (64.3%) were diagnosed with osseous alterations in at least one TMJ. 52 (24.4%) had unilateral and 85 (39.9%) had bilateral degenerative changes. Distribution by gender, please see Table 1.

**Table 1:** Prevalence of cone beam computed tomography osseous temporomandibular joint alterations

TMJ osseous alterations	Male (*n* = 78)	Female (*n* = 135)	Total (*n* = 213)
Bilateral (*n*) (%)	26 (12.2)^a^	59 (27.7)^a^	85 (39.9)
Unilateral (*n*) (%)	18 (8.5)^b^	34 (16.0)^b^	52 (24.4)
At least one (*n*) (%)	43 (20.2)^c^	94 (44.1)^c^	137 (64.3)

*n,* number of subjects; (%), percent; ^a^, *p* 0.136; ^b^, *p* 0.730; ^c^, *p* 0.033 (Chi square = 4.530, df = 1)

Table 2 presents the spectrum of osseous changes of the condyle and the fossa in at least one TMJ. Condylar erosions were the most prevalent alterations present in 88 (41.3 %) of subjects, followed by condylar flattening (60 (28.2 %)), osteophyte formation (52 (24.4 %)), and sclerosis (34 (16 %)). Statistically significant gender differences were seen in the prevalence of condylar and fossa sclerosis.

**Table 2:** Distribution of cone beam computed tomography osseous alterations in at least one temporomandibular joint

TMJ osseous alterations	Male (*n* = 78)	Female (*n* = 135)	Total (*n* = 213)
Condyle			
Erosion (*n*) (%)	28 (35.9)	60 (44.4)	88 (41.3)
Osteophyte (*n*) (%)	18 (32.1)	34 (25.2)	52 (24.4)
Flattening (*n*) (%)	20 (25.6)	40 (29.6)	60 (28.2)
Sclerosis (*n*) (%)	6 (7.7)^a^	28 (20.7)^a^	34 (16.0)
Resorption (*n*) (%)	2 (2.6)	8 (6.2)	10 (4.7)
Subchondral Cyst (*n*) (%)	2 (2.6)	6 (4.4)	8 (3.8)
Fossa			
Sclerosis (*n*) (%)	2 (2.6)^b^	15 (11.1)^b^	17 (8.0)

*n,* number of subjects; (%), percent; ^a^, *p* 0.136; ^b^, *p* 0.730; ^c^, *p = *0.033 (Chi-square = 4.530, df = 1)

### Orthodontic retainer wires

Fixed retainer wires were present in 55 subjects (25.8%). Table 3. compares the prevalence of CBCT osseous alterations between individuals with and without retainer wires. Statistical analysis revealed a significant difference in the prevalence of condylar erosions and condylar flattening between the two subgroups. Furthermore, statistically significant gender differences in the presence of condylar sclerosis (in both subgroups) and in the presence of condylar flattening (in the non-retainer wire group) were observed.

**Table 3.**
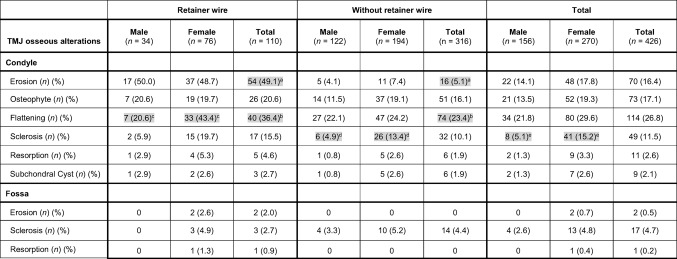
Prevalence of cone beam tomography osseous alterations of temporomandibular joints (n = 426) in subjects with and without orthodontic retainer wires

*TMJ*, temporomandibular joint; *n*, number of temporomandibular joints; (%), percent; ^a^, *p* < 0.001 (Chi-square = 115.184, df = 1); ^b^, *p* = 0.008 (Chi-square = 6.977, df = 1); ^c^, *p* = 0.021 (Chi-square = 5.292, df = 1); ^d^, *p* = 0.015 (Chi-square = 5.924, df = 1); ^e^, *p* = 0.002 (Chi-square = 9.824, df = 1). Statistically significant differences are marked in grey.

Binary multivariate logistic regression analysis of influencing factors of CBCT condylar erosions, presenting the most prevalent osseous alterations, showed that odds to have condylar erosions were 4.046 times higher in individuals with retainer wires than in those without wires (*p* < 0.001) (Table 4).

**Table 4:** Results of binary multivariate logistic regression analysis of influencing factors of cone beam computed tomography condylar erosion alterations in at least one temporomandibular joint of subjects (*n* = 213)

Variables	*B*	*S.E.*	Wald statistic	*p*	*OR*	95% *CI*
Age	−0.085	0.044	3.789	0.052	0.919	0.844–1.001
Gender	−0.219	0.311	0.879	0.349	0.747	0.406–1.374
ORW	1.398	0.338	17.134	< 0.001	4.046	2.087–7.841

*ORW,* orthodontic retainer wire; *B,* unstandarlized regression weight; *S.E,* standard error; *p, *probability of type I error; *OR, *odds atio; *CI, *confidence interval

Binary logistic regression analysis of influencing factors (age, gender, orthodontic retainer wire) revealed an odds ratio of 0.916 (*p* = 0.804) to have flattening alterations in at least one temporomandibular joint of subjects (*n* = 213).

## Discussion

The aim of this retrospective study was to assess the prevalence of degenerative osseous TMJ findings in a consecutive sample of young adults, who had skull CBCT prior to the surgical removal of wisdom teeth.

We found in our study sample a prevalence of 64.3% alterations in at least one TMJ. The unexpectedly high rate of degenerative TMJ findings in individuals aged 23.9 ± 3.5 years is comparable to the prevalence of TMJ bone alterations that Dumbuya et al. (2020) assessed in older adults (*n* = 137, age 73.35 ± 6.28 years) without previous TMJ surgery or trauma, who had skull CBCT scans for unrelated indications regardless of TMD status. Subchondral cysts, osteophytes, flattening, sclerosis, or erosions were detected in 65.7% of subjects in at least one TMJ [14]. Subjects with TMJ bone changes had a higher prevalence of osteoporosis/osteopenia and connective tissue disorders than subjects with normal TMJs.

Singh et al. (2021) retrospectively evaluated incidental findings of the TMJ region in 1,850 randomly selected CBCT scans that were taken for orthodontic evaluation, implant pre-assessment, difficult tooth extractions, dento-alveolar pathologies, endodontic purposes, or cleft palates or lips. A prevalence of 59.89% of incidental TMJ findings was noted (35.6% of which flattening of the condyle, 28.3% soft tissue calcifications, 13.8% cysts). Age-group-wise distribution of incidental findings (0 to 10, 11 to 20, 21 to 30, 31 to 40, 41 to 50, 51 to 60, and 61 to 70 years) revealed the highest prevalence of incidental findings in 21 to 30- (23.64%) and 31 to 41-year olds (39.53%) [15].

In the course of another retrospective study, CBCT scans of 300 patients (mean age 46.73, range 18 to 74 years), taken for implant treatment planning, were analyzed with regard to incidental findings of the categories maxillary sinus pathologies, TMJ findings, dentoalveolar findings, and soft-tissue calcifications [16]. 49.3% of the patients displayed at least one TMJ incidental finding in at least one joint, most frequently a combination of erosion and osteophyte formation. 29.5% of TMJs were diagnosed with erosions, 21.3% with osteophytes, 7.2% with sclerosis, 7.7% with flattening, and 4.3% with a bifid condyle.

Price et al. (2012) evaluated 272 consecutive CBCT scans of a similar age group (mean patient age 49.3, range 9 to 80 years) with respect to incidental findings (defined as findings that were unrelated to the primary purpose of the scan) [17]. Of all incidental findings (*n* = 881), airway findings (35%), soft tissue calcifications (20%), bone alterations (17.5%), and TMJ changes (15%) were the most prevalent. Condyle flattening occurred in 12.1%, condylar erosion in 8.8%, subchondral pseudocysts in 6.3%, osteophytes in 4.44%, subchondral sclerosis in 2.9%, and bifid condyle shape in 0.4% of patients.

Lopes et al. analyzed 150 CBCT examinations of patients (mean age 37 ± 18.3, range 8 to 91 years), taken for indications unrelated to TMD (implant planning, tumor or cyst evaluation or monitoring, third molar pre-operative assessment, unerupted tooth assessment, supernumerary tooth location, and others) [18]. CBCT scans were, according to the size of the field of view, divided into three groups (6-cm maxilla, 6-cm mandible, 13-cm maxilla/mandible) and evaluated with respect to incidental findings in six areas, one of which the TMJ. In the field of view encompassing the maxilla and the mandible, osteophyte formation was seen in 5%, flattening of the condyle in 15.3%, and a bifid condyle in 0.3% of patients. While authors judged a bifid condyle as an anatomical variant that requires no treatment, osteophyte formation and condyle flattening were regarded as need to follow-up.

A late-breaking systematic review on incidental findings from CBCT of the head/neck region for diagnostic reasons in children and adolescents included ten studies covering 1,818 patients (average age 12.3 years) [19]. Incidental findings were most often related to airways (63.7%), bone (23.6%), spine (26.2%), teeth (19.2%), and TMJ (3.8%; 1.2% of which osteoarthritis). Authors ascertained several methodological shortcomings (incomplete reporting of patient- or CBCT-related details, incomplete categorization and reporting on the severity of findings, small sample sizes, and research transparency issues). They concluded that incidental findings are often seen in CBCT scans of children or adolescents and that any CBCT image should be adequately assessed for incidental findings by either a specialist oral and maxillofacial radiologist or a dentist with appropriate training and experience.

Altogether, in our study population of young adults, prevalence of osseous TMJ changes (64.3% in at least one TMJ) rather compared to results of previous CBCT studies in older adults, whereas it exceeded the prevalence of TMJ alterations previously found in adolescents or young adults.

Görürgöz et al. (2023) correlated degenerative changes in the condylar surface with patient age in a sample of 258 individuals (mean age 39.81 ± 15.6 years; CBCTs taken for various reasons) and found an increased risk of radiographically detectable degenerative alterations with increasing age [20].

Sing et al. (2021), in contrast, assessed the highest prevalence of incidental osseous TMJ findings in age groups from 20 to 40 years [15].

Wiese et al. (2018) evaluated TMJ tomograms of 204 patients with TMJ symptoms and compared expected (according to clinical examination using the Research Diagnostic Criteria for TMDs (RDC/TMD [1]) and actual radiographic findings. Tomography often revealed unexpected findings and the number of radiographic findings was mostly underestimated [21]. Other researchers have also claimed poor correlation between osseous TMJ alterations and the severity of symptoms in TMJ osteoarthritis [22–24]. Clinical signs seem to not necessarily correspond with radiographic diagnoses, and the clinical relevance of radiographic findings is questionable, as is the strict RCD/TMD categorization of degenerative osseous TMJ findings as osteoarthritis (in association with pain) or osteoarthrosis (in absence of pain). It seems unlikely that two thirds of our random sample of young study participants would suffer from progressive TMD such as osteoarthritis or osteoarthrosis. Unfortunately, within the retrospective study design, neither information on study participants’ clinical TMD status nor data on the type of occlusion, e.g. class I, II, or III, open bite, crossbite, etc., or mode of previous orthodontic treatment were available.

Another limitation of our study is a lack of follow-up imaging, which would allow the assessment of persistence, change, decrease, or increase of radiological signs. Albeit, the obtainment of ethical approval for repeated X-ray exposure for mere research reasons is quite out of the question. Degenerative osseous TMJ changes reflect an age-related bone remodeling process [9] and any isolated TMJ image just presents a snapshot in time.

One limitation is owed to the inadequacy to assess all aspects of degenerative bony changes that define the TMJ articular surface destruction by CBCT alone. The diagnostic accuracy of CBCT in detecting TMJ bony disorders has been confirmed by two recent systematic reviews in vitro and in vivo [25, 26]. CBCT effectively rules out false positive results, while there remains a risk to miss true positives (pooled specificity 0.93; sensitivity 0.54) [25]. The additional use of Magnetic resonance tomography (MRT) might have facilitated an even more comprehensive diagnosis of osseous TMJ alterations [27].

One fundamental limitation concerns the aspect that most clinical experience is commonly restricted by the limitation of observer variations, which tend to have a significant impact on the diagnosis process. The possibility of rater bias relating to the clinician who assessed the CBCT variables must also be considered. Observer performance can be affected by various factors such as training, quality of an image, and the specific criteria for interpretation. This study used well-defined criteria in the interpretation of CBCT variables, and the CBCT images used were of high quality. The CBCT images of the TMJ were reported based on intra-observer reliability that was within the accepted limits for a diagnostic study. However, no measurement of inter-observer reliability was taken. As a result, overrating may have caused some of the variations of erosion reported in this study. Consequently, overestimation of the relevance of these factors to the described groups may have occurred. With regard to the diagnosis of condylar erosion, the radiologist may have been influenced by the state of the other joint components.

Our study may serve as incentive for other clinicians/radiologists to assess degenerative TMJ alterations in the general population, whenever CBCT scans of the TMJ are taken (for reasons unrelated to TMD), preferably in correlation with clinical TMJ findings, occlusal classification and, if applicable, by use of a prospective long-term study design to follow-up clinical and/or radiological signs.

In the light of the broad variety of dental malocclusions (requiring therapy) and the wide spectrum of orthodontic treatment modalities and appliances, the correlation of previous orthodontic treatment (indicated by fixed retainer wires) with the presence of radiographic TMJ findings seems somewhat illegitimate. Moreover, authors are aware of bias, since the absence of an orthodontic retainer wire does not exclude previous orthodontic treatment. Some recent CBCT studies have correlated the presence of osseous TMJ alterations with occlusion classes. Results are widespread and controversial.

Loiola at al. (2023) assessed CBCT scans of 55 patients with class I, II, and III occlusion to measure (among other parameters) condylar surface alterations [28]. In condylar surface changes, no statistically different values for different occlusion classes were detected.

Oliveira et al. (2024) investigated the prevalence of condylar morphological changes in 70 individuals (aged between 25 and 50 years) with class II malocclusions with respect to different vertical growth patterns [29]. They found a higher relative prevalence of bone changes in dolichofacial individuals with flattening (62%), sclerosis (44%), and subchondral bone cyst (20%). Erosion and osteophytes prevailed in mesofacial (39%), and brachyfacial individuals (32%), respectively. There was altogether no statistically significant difference in the prevalence of degenerative changes between the vertical skeletal patterns. Flattening was the most prevalent change, whereas subchondral bone cyst was the least prevalent among the three groups studied.

A cross-sectional retrospective Chinese study in CBCT images of 90 adolescents investigated fossa and condyle shape and found that the glenoid fossa was wider and shallower in individuals with skeletal Class III malocclusion with mesocephalic and dolichocephalic profiles than in class I controls [30].

Another CBCT study that measured condyle and fossa dimensions in 92 individuals with class II division 1 skeletal relationship and 96 individuals with class I (controls) (aged between 15 and 65 years), assessed smaller condyle dimension values in class II/1 than in class I individuals [31]. Authors postulated a possible decrease in the dimensions of the eminence and the condyle with an increasing ANB angle.

Thus, not only (mal-) occlusion classes but also the vertical dimension impact condyle and fossa morphology by varying force magnitudes, directions, and distributions onto TMJ structures [29, 31]. The complexity of TMJ hard and soft tissue structures and the multitude of possible pathogenetic factors seem to render a systematic correlation between occlusion and degenerative TMJ alterations difficult, not to speak of effects of orthodontic measures.

Very little literature referring to the impact of orthodontic treatment onto osseous TMJ structural changes (such as flattening, erosion, osteophyte formation, sclerosis, or resorption) is available. A systematic review (including eleven papers reporting eight clinical trials involving altogether 377 patients (average age 10.3 years)) analyzed the radiologic effects (measured with MRI, CT, or CBCT) of functional appliance class II treatment (compared to no treatment) onto the TMJ [32]. Functional appliance treatment was associated with increased condylar width, decreased anterior joint space, increased superior joint space, or vertical displacement of the glenoid fossa. Authors concluded that functional appliance treatment is associated with positional and skeletal alterations of the TMJ in the short term compared to untreated controls. However, the clinical relevance of these changes remains unclear, while the quality of existing evidence is low due to methodological issues of existing studies [32].

Our study may serve to draw orthodontists’ attention to the high prevalence of degenerative TMJ alterations in young persons that should be considered in treatment planning. It seems conceivable that orthodontic treatment may induce or promote not only remodeling [33] but also (transient?) osseous alterations in adolescents’ or young adults’ TMJs. This aspectcalls for further investigation in future studies.

## Conclusion

Degenerative osseous TMJ changes are a frequent finding in young adults.

Future studies need to clarify the clinical relevance of osseous TMJ alterations and to investigate their pathogenesis and pro- or regression in connection with diverse malocclusions and orthodontic measures.

## Data Availability

All data generated or analyzed during this study are not publicly available due to ethical and confidentiality reasons. The data will only be shared in aggregate form as presented in the figures and tables.
